# Sudden Death by Pulmonary Thromboembolism due to a Large Uterine Leiomyoma with a Parasitic Vein to the Mesentery

**DOI:** 10.1155/2014/181265

**Published:** 2014-12-21

**Authors:** Varsha Podduturi, Danielle R. Armstrong-Briley, Joseph M. Guileyardo

**Affiliations:** Department of Pathology, Baylor University Medical Center, Dallas, TX, USA

## Abstract

The pathophysiology of venous thrombosis is classically attributed to alterations in one or more components of Virchow's triad: hypercoagulability, stasis, and damage to the vascular endothelium. Deep vein thrombosis (DVT) may lead to pulmonary thromboembolism (PE), and the latter is culpable for many deaths annually in the United States; however, DVT as a complication of uterine leiomyoma has rarely been reported. We report a case of a 57-year-old woman whose death was due to a large pedunculated subserosal leiomyoma externally compressing the pelvic veins resulting in stasis and venous thrombosis leading to fatal PE. The association of large pelvic masses with venous thrombosis has clinical implications, since prophylactic surgery could be life-saving.

## 1. Introduction

Modifications to one or more components of the classic Virchow triad (stasis, endothelial damage, and hypercoagulability) increase the probability of DVT. A venous thrombus may subsequently dislodge and embolize to the pulmonary vasculature where it may be fatal. Clinically, it is important to identify patients at risk for DVT, so appropriate prophylaxis can be provided. However, in fatal cases it is important to identify the source of a thromboembolus at autopsy as well as to search for risk factors of hypercoagulability when possible since the underlying cause may vary. Diverse causes have been associated with DVT including surgical procedures, pregnancy, disseminated malignancy, immobility, and inherited and acquired hypercoagulable states; however, DVT as a complication of uterine leiomyomata is not common [1–14]. We present the clinical, pathologic, and autopsy findings of a fatal case of a large uterine leiomyoma causing pelvic venous stasis resulting in bilateral DVT and PE.

## 2. Case Presentation

A 57-year-old unemployed African American woman with poorly controlled hypertension, congestive heart failure, and polysubstance abuse (including crack-cocaine) presented after one month of progressive nonproductive cough and dyspnea and two weeks of anorexia and fatigue. EMS was summoned to her home after she complained of increasing dyspnea. Her oxygen saturation measured 70% on room air. She was taken to Baylor University Medical Center at Dallas and admitted. Blood pressure was 187/99 mmHg, heart rate 84 beats per minute, respirations 28 breaths per minute, and temperature 98.0 degrees Fahrenheit. Initial laboratory values were significant for hemoglobin 12.4 g/dL; platelets 254 K/uL, creatinine of 1.7 mg/dL; glucose 237 mg/dL; and B-type natriuretic peptide 394 pg/mL (reference range 0–100 pg/mL). Liver function tests included aspartate aminotransferase of 103 U/L (reference range 5–35 U/L); alkaline phosphatase 129 U/L (reference range 38–126 U/L); and albumin 2.9 g/dL (reference range 3.5 to 4.8 g/dL). She had no recent long trips and she was not taking any current medications.

**Figure 1:**
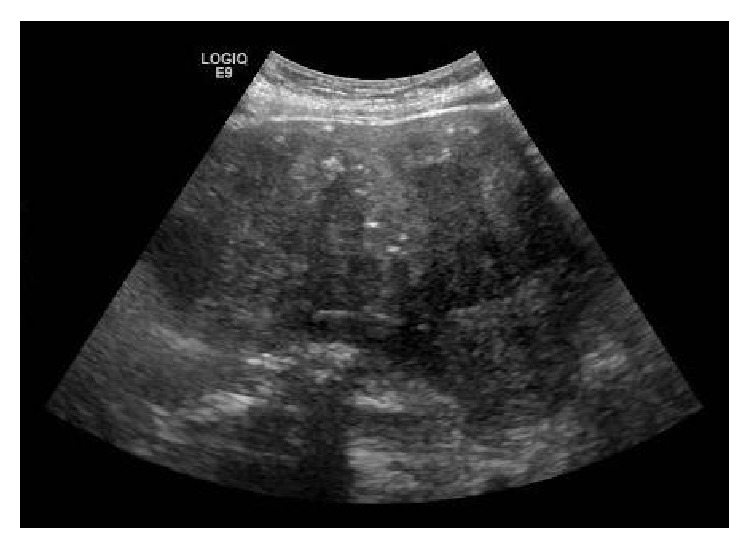
Ultrasound image showing a large pelvic mass.

Physical examination found diffuse rales, rhonchi, wheezing, and generalized abdominal tenderness. No lower extremity swelling was noted. Her body mass index was 31.8 kg/m^2^. Chest X-ray showed cardiomegaly and bilateral pulmonary infiltrates suggesting pneumonia. Pelvic sonogram found a large mass suggestive of a fibroid uterus, posterosuperior to the bladder (Figure 1). A nasal swab was positive for H1N1 influenza by PCR. She was placed on empiric antibiotics and BiPAP; however, her respiratory function continued to decline. Thirteen days after admission, she experienced sudden cardiopulmonary arrest and died.

**Figure 2:**
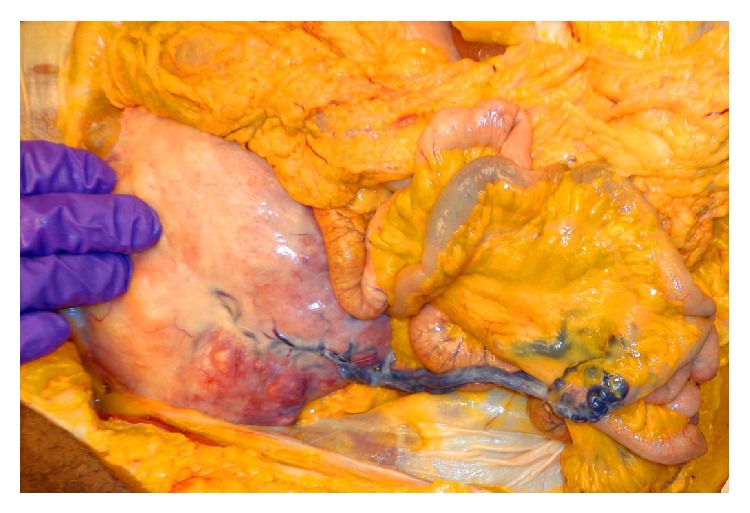
Vessel from the leiomyoma parasitizing to the mesentery.

Autopsy found a normally developed, moderately obese woman. An oral endotracheal tube was well positioned. A 1275-gram, 14.8 × 14.2 × 9.4 cm subserosal pedunculated tan-white uterine nodule extended from the fundus and filled the entire pelvis with extension into the lower abdominal cavity. A vein from the surface of the nodule anastomosed with an adjacent small bowel mesenteric vein (Figure 2). The myometrium also contained smaller intramural and subserosal nodules ranging in size from 0.7 cm to 2.2 cm in diameter. Cut sections of all nodules were whorled and tan-white. Multiple laminated and focally adherent hilar and peripheral intravascular thrombi were present in all lobes of both lungs. Posterior leg dissection found thrombi in bilateral posterior tibial veins and deep gastrocnemius veins.

**Figure 3:**
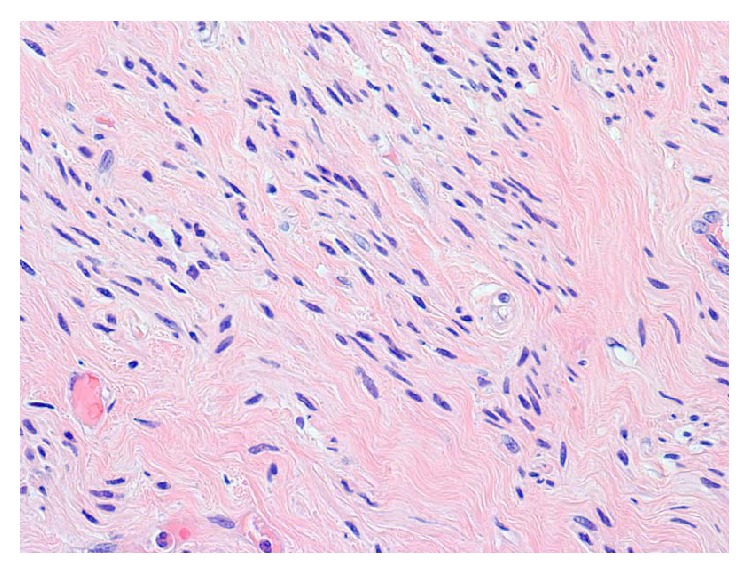
Sections from large and small nodules find smooth muscle fascicles (H&E 400x).

**Figure 4:**
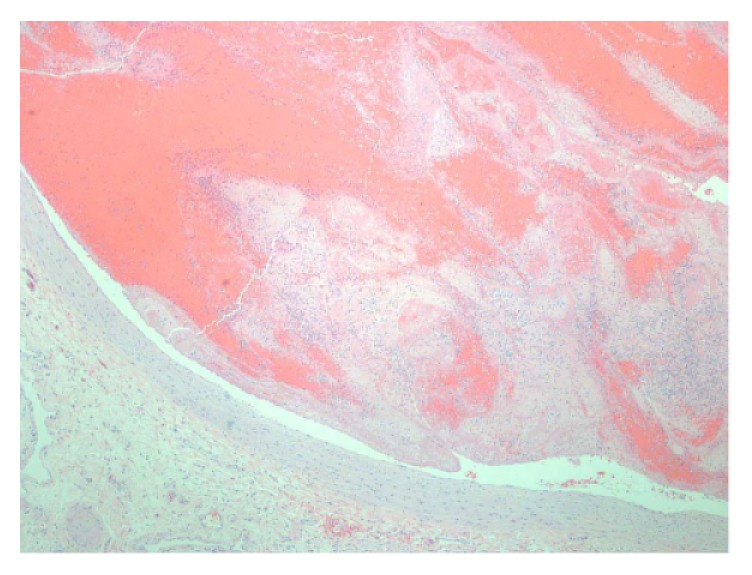
Laminated thrombus within the pulmonary artery (H&E 100x).

**Figure 5:**
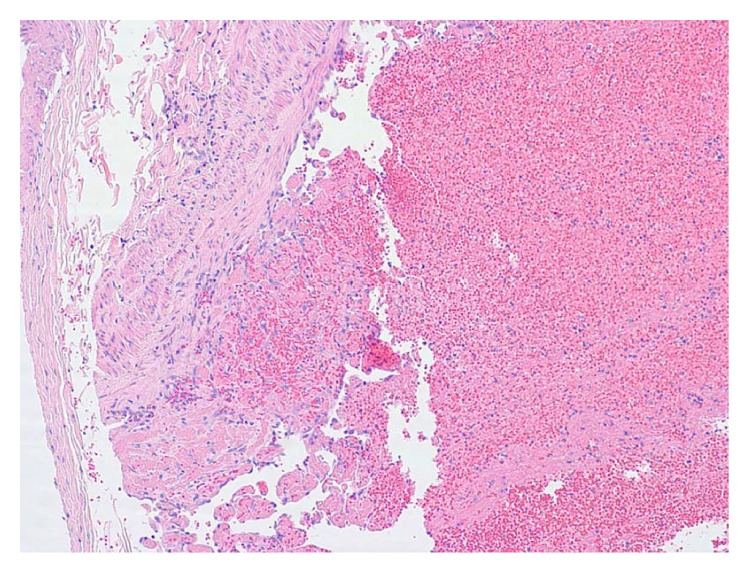
Focal endothelial organization within the left leg vein (H&E 100x).

Microscopically, the myometrial nodules consisted of smooth muscle fascicles with areas of interstitial hyalinization (Figure 3). No coagulation necrosis or mitotic figures were identified; and the masses were classified as leiomyomas. Sections from the leg veins and lungs showed laminated thrombi (Figure 4) with focal endothelial organization (Figure 5). Additional autopsy findings included cardiomegaly (485 grams) with atherosclerotic and hypertensive cardiovascular disease and diffuse alveolar damage. Postmortem studies to investigate for hypercoagulability, including MTHFR (methylenetetrahydrofolate reductase), prothrombin G20210A, and factor V Leiden mutations, were performed and were negative.

The cause of death was pulmonary thromboembolism due to DVT associated with a very large uterine leiomyoma and probably contributory to death was obesity. The manner of death was natural.

## 3. Discussion

Uterine leiomyomas are the most common pelvic tumors in women, occur in 20–30% of women over 30 years of age [15], and may be subserosal, submucosal, or intramural. Parasitic leiomyoma is rare and occurs when a pedunculated subserosal leiomyoma becomes detached from the uterus and is supplied by nonuterine vasculature [16]. The most common symptom of uterine leiomyoma is menorrhagia and less frequently pelvic pain or pressure [15]; and the rate of growth is affected by estrogen, growth hormone, and progesterone [15]. Treatment options include gonadotropin-releasing hormone therapy, uterine artery embolization, myomectomy, myolysis, and hysterectomy [15]. Despite their frequency, leiomyomas associated with DVT are not common [1–14].

As stated above venous thrombosis may be attributed to alterations in Virchow's triad and those at risk may have one or more abnormalities in any of the three elements. Hemostasis as a result of immobility is a common risk factor for DVT and PE [17, 18], and causes of immobility include long distance travel, surgery, prolonged hospitalization, and spinal cord injuries (quadriplegia, paraplegia) [17–21].

Hypercoagulable states may be inherited or acquired. Genetic abnormalities include protein C and protein S deficiencies, antithrombin III deficiency, and factor V Leiden, prothrombin, and MTHFR mutations [17, 20, 22]. Other inherited thrombophilias include dysfibrinogenemias, deficiency of heparin cofactor II, and abnormal thrombomodulin. [23]. Use of oral contraceptives, hormone replacement therapy, sepsis, pregnancy, and malignancy are acquired states that also confer an increased risk of venous thrombosis [17, 20–22]. Additional acquired states include the antiphospholipid antibody syndrome (caused by lupus anticoagulant, anti-cardiolipin, and anti-*β*_2_-glycoprotein-I antibodies), heparin induced thrombocytopenia, and paroxysmal nocturnal hemoglobinuria [24, 25].

Direct endothelial damage is probably less common than the aforementioned factors and is often due to trauma or recent surgical intervention of the lower limbs [17, 20].

Additional risk factors for DVT include decreased cardiopulmonary reserve (including congestive heart failure and cor pulmonale with interstitial lung diseases), general debility [17], and decreased mobility with increasing age [18, 20]. In our case the large uterine leiomyoma most likely compressed the pelvic veins resulting in stasis and thrombosis in the leg veins and fatal pulmonary thromboembolism.

Also fascinating in our case is the tumor acquired parasitic blood supply via a tortuous vessel from the adjacent bowel mesentery. Parasitism of the nearby vasculature by adrenal, renal, and hepatic neoplasms [26] and uterine leiomyomata [27] has been attributed to vascular adhesions between the mass and the surrounding structures [26].

A fatal outcome in this case may have been avoided by prophylactic anticoagulation. Unfortunately, she received none, although she was bedridden for several days. Additionally, prophylactic hysterectomy may have been life-saving. This case highlights the fact that potential complications of large uterine leiomyomas, or any large pelvic masses, are profound and considerations of prophylactic therapy are warranted.

## References

[1] Dekel A., Rabinerson D., Dicker D., Ben-Rafael Z. (1998). Thrombosis of the pelvic veins associated with a large myomatous uterus. *Obstetrics and Gynecology*.

[2] Chong Y. S., Fong Y. F., Ng S. C. (1998). Deep vein thrombosis in patients with large uterine myomata. *Obstetrics and Gynecology*.

[3] Nishikawa H., Ideishi M., Nishimura T., Kawamura A., Kamochi H., Tahara H., Tsuchiya Y., Shirai K., Okabe M., Arakawa K. (2000). Deep venous thrombosis and pulmonary thromboembolism. *Angiology*.

[4] Falcone M., Serra P. (2005). Massive pulmonary embolism in a woman with leiomyomatous uterus causing pelvic deep venous thrombosis. *Annali Italiani di Medicina Interna*.

[5] Yonezawa K., Yokoo N., Yamaguchi T. (1999). Effectiveness of an inferior vena caval filter as a preventive measure against pulmonary thromboembolism after abdominal surgery. *Surgery Today*.

[6] Stanko C. M., Severson M. A., Molpus K. L. (2001). Deep venous thrombosis associated with large leiomyomata uteri: a case report. *Journal of Reproductive Medicine*.

[7] Phupong V., Tresukosol D., Taneepanichskul S., Boonkasemsanti W. (2001). Unilateral deep vein thrombosis associated with a large myoma uteri. A case report. *Journal of Reproductive Medicine for the Obstetrician and Gynecologist*.

[8] Hawes J., Lohr J., Blum B., Bhati A., Bhaskaran J., Engel A. (2006). Large uterine fibroids causing mechanical obstruction of the inferior vena cava and subsequent thrombosis: a case report. *Vascular and Endovascular Surgery*.

[9] Bonito M., Gulemì L., Basili R., Brunetti G., Roselli D. (2007). Thrombosis associated with a large uterine myoma: case report. *Clinical and Experimental Obstetrics and Gynecology*.

[10] Khilanani R., Dandolu V. (2007). Extensive iliac vein thrombosis as a rare complication of a uterine leiomyoma: a case report. *Journal of Reproductive Medicine for the Obstetrician and Gynecologist*.

[11] Tanaka H., Umekawa T., Kikukawa T., Nakamura M., Toyoda N. (2002). Venous thromboembolic diseases associated with uterine myomas diagnosed before hysterectomy: a report of two cases. *Journal of Obstetrics and Gynaecology Research*.

[12] Srivatsa A., Burdett J., Gill D. (2005). A 35-year-old woman with uterine fibroids and multiple embolic strokes. *Neurology*.

[13] Srettabunjong S. (2013). Systemic thromboembolism after deep vein thrombosis caused by uterine myomas. *The American Journal of Forensic Medicine and Pathology*.

[14] Rosenfeld H., Byard R. W. (2012). Lower extremity deep venous thrombosis with fatal pulmonary thromboembolism caused by benign pelvic space-occupying lesions—an overview. *Journal of Forensic Sciences*.

[15] Evans P., Brunsell S. (2007). Uterine fibroid tumors: diagnosis and treatment. *American Family Physician*.

[16] Kho K. A., Nezhat C. (2009). Parasitic myomas. *Obstetrics & Gynecology*.

[17] Kroegel C., Reissig A. (2003). Principle mechanisms underlying venous thromboembolism: epidemiology, risk factors, pathophysiology and pathogenesis. *Respiration*.

[18] Heit J. A. (2008). The epidemiology of venous thromboembolism in the community. *Arteriosclerosis, Thrombosis, and Vascular Biology*.

[19] Gavish I., Brenner B. (2011). Air travel and the risk of thromboembolism. *Internal and Emergency Medicine*.

[20] Esmon C. T. (2009). Basic mechanisms and pathogenesis of venous thrombosis. *Blood Reviews*.

[21] Ho W. K. (2010). Deep vein thrombosis—risks and diagnosis. *Australian Family Physician*.

[22] Previtali E., Bucciarelli P., Passamonti S. M., Martinelli I. (2011). Risk factors for venous and arterial thrombosis. *Blood Transfusion*.

[23] Rao A. K., Sheth S., Kaplan R. (1997). Inherited hypercoagulable states. *Vascular Medicine*.

[24] Ziakas P. D., Poulou L. S., Pomoni A. (2008). Thrombosis in paroxysmal nocturnal hemoglobinuria at a glance: a clinical review. *Current Vascular Pharmacology*.

[25] Magnani H. N. (1993). Heparin-induced thrombocytopenia (HIT): an overview of 230 patients treated with orgaran (Org 10172). *Thrombosis and Haemostasis*.

[26] Sprayregen S. (1973). Parasitic blood supply of neoplasms. *Radiology*.

[27] Makris A., Talmor A., Moyle P., Majmudar T., Abdel-Rahman H. (2012). Parasitic fibroid and pseudoMeigs' syndrome: co-existence of two rare entities. *Journal of Obstetrics and Gynaecology*.

